# Maternal hepatic immunology during pregnancy

**DOI:** 10.3389/fimmu.2023.1220323

**Published:** 2023-06-30

**Authors:** Ling Yang, Yao Meng, Yuxiang Shi, Hongxu Fang, Leying Zhang

**Affiliations:** School of Life Sciences and Food Engineering, Hebei University of Engineering, Handan, China

**Keywords:** hormone, immunology, liver, pregnancy, growth factor

## Abstract

The liver plays pivotal roles in immunologic responses, and correct hepatic adaptations in maternal immunology are required during pregnancy. In this review, we focus on anatomical and immunological maternal hepatic adaptations during pregnancy, including our recent reports in this area. Moreover, we summarize maternal pregnancy-associated liver diseases, including hyperemesis gravidarum; intrahepatic cholestasis of pregnancy; preeclampsia, specifically hemolysis, elevated liver enzymes, and low platelet count syndrome; and acute fatty liver of pregnancy. In addition, the latest information about the factors that regulate hepatic immunology during pregnancy are reviewed for the first time, including human chorionic gonadotropin, estrogen, progesterone, growth hormone, insulin like growth factor 1, oxytocin, adrenocorticotropic hormone, adrenal hormone, prolactin, melatonin and prostaglandins. In summary, the latest progress on maternal hepatic anatomy and immunological adaptations, maternal pregnancy-associated diseases and the factors that regulate hepatic immunology during pregnancy are discussed, which may be used to prevent embryo loss and abortion, as well as pregnancy-associated liver diseases.

## 1 Introduction

The liver contains macrophages (resident Kupffer cells, KCs), natural killer (NK) cells, natural killer T (NKT) cells, and reticuloendothelial cell networks that act as immune sentinels and effector cells to detect and capture circulating pathogens (1). There are physiological, hormonal and immunological adaptations in the mother during pregnancy, and the decidua and placenta form key immunological barriers to protect the fetus and promote its growth (2). However, the immunological responses at the receptive maternal-fetal interface are highly dynamic and not simply suppressed, and a tolerogenic maternal immune system is required for a successful pregnancy (3). Furthermore, peripheral maternal immune system components are dynamically modulated during normal pregnancy, and significant systemic immunological adaptation is necessary to avoid detrimental immune responses against the allogeneic fetus (4). As a frontline immune tissue, the liver can mount a rapid and robust immune response, and portal blood can transport a large number of foreign but harmless molecules (5). In addition, the liver works as a lymphoid organ to facilitate tolerance rather than immunoreactivity, which is essential for fetal immune tolerance (6).

Successful pregnancy is dependent on correct hepatic adaptations in maternal immunology. However, there has not been a review that focuses on maternal liver immunology during pregnancy. In this review, we first focus on our current understanding of anatomical and immunological maternal hepatic adaptations, as well as pregnancy-associated hepatic diseases. Then, we summarize the factors that regulate hepatic immunology during pregnancy. It is essential to avoid detrimental immune responses against the allogeneic fetus and prevent maternal hepatic diseases related to pregnancy.

## 2 Hepatic cellular anatomy

**Figure 1:**
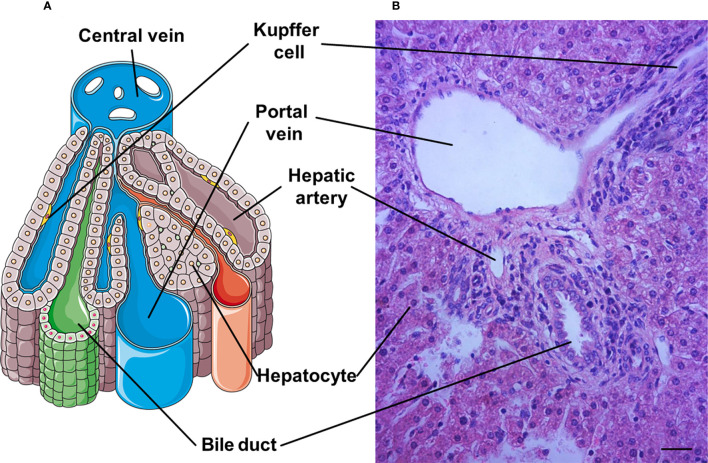
A representative hepatic lobule. **(A)** The representative diagram of a hepatic lobule. **(B)** Hepatic tissue stained by haematoxylin and eosin includes a representative histological image of hepatic lobule. A portal triad is a component of the hepatic lobule, consists of proper hepatic artery, hepatic portal vein, small bile duct and hepatic sinusiod. Branches of the portal vein and the hepatic artery merge upon entry into the liver lobule at the portal field for blood supply of the liver, and exits at the central vein. Bar = 50 µm.

The microscopic architecture of the liver is similar in all mammals, although there are obvious differences in the hepatic lobation and gallbladder between rodents and humans. The internal structure of the liver consists of small hexagonal lobule that is the functional structural unit of the liver, and includes hepatocyte plates and a central vein (7). The terminal twigs of the portal vein, hepatic artery and bile duct surround the central vein at the portal field (8). In addition, the hepatic lobule contains the portal vein, hepatic artery, bile duct and hepatic sinusoid (**Figure 1**). The liver is composed of hepatocytes, biliary epithelial cells, stellate cells, KCs, and liver sinusoidal endothelial cells (LSECs) that cooperatively regulate hepatic function at multiple levels (7).

## 3 Anatomical maternal hepatic adaptations

There is gestational hepatomegaly to meet the increasing metabolic demands during pregnancy. The size of the liver increases, and is proportional to the increase in body weight of pregnant rats. Furthermore, liver growth during pregnancy is related to maternal body weight gain, fetal number and hepatocyte hypertrophy, which is induced by circulating reproductive hormones in rodents (9). Moreover, the increase in liver size with pregnancy is regulated by the reproductive state and fetal growth during pregnancy in women (10). In addition, pregnancy-induced hepatomegaly is initiated following implantation, peaks at parturition and is associated with interleukin (IL)-6, tumor necrosis factor (TNF)-α, c-JUN and IL-1β, and the activation of hepatic signal transducer and activator of transcription (STAT) 3, β-catenin and epidermal growth factor receptor (11).

It has been reported that portal venous and total liver blood flows are significantly increased after 28 weeks gestation, but the hepatic arterial blood flow shows no significant changes during pregnancy in humans (12). Furthermore, the resistive index in the common hepatic artery decreases during the third trimester of pregnancy, and this decrease is related to systemic arterial vasodilatation during normal pregnancy (13). Moreover, the flow velocity of the portal vein is higher in the first trimester than in the second and third trimesters and can be used as an indicator of physiological alterations related to pregnancy (14). However, there may be more cytokines and growth factors related to anatomical maternal hepatic adaptations during pregnancy, and so further studies on this area may be needed.

## 4 The immunological structure of the liver

Two blood supplies in the liver, the hepatic artery and the portal vein, carry potential pathogens, microbe-derived molecules, nutrients, and old, oxidized, or damaged molecules for clearance (5). The small diameter of the sinusoids, minimal systemic venous pressure and perturbations in sinusoidal flow slow blood flow in the liver, which lengthens the time during which the liver can facilitate intimate interactions between resident immune cells and nonhematopoietic hepatic cells, and ultimately, the blood leaves the liver *via* the central hepatic veins (15).

**Figure 2:**
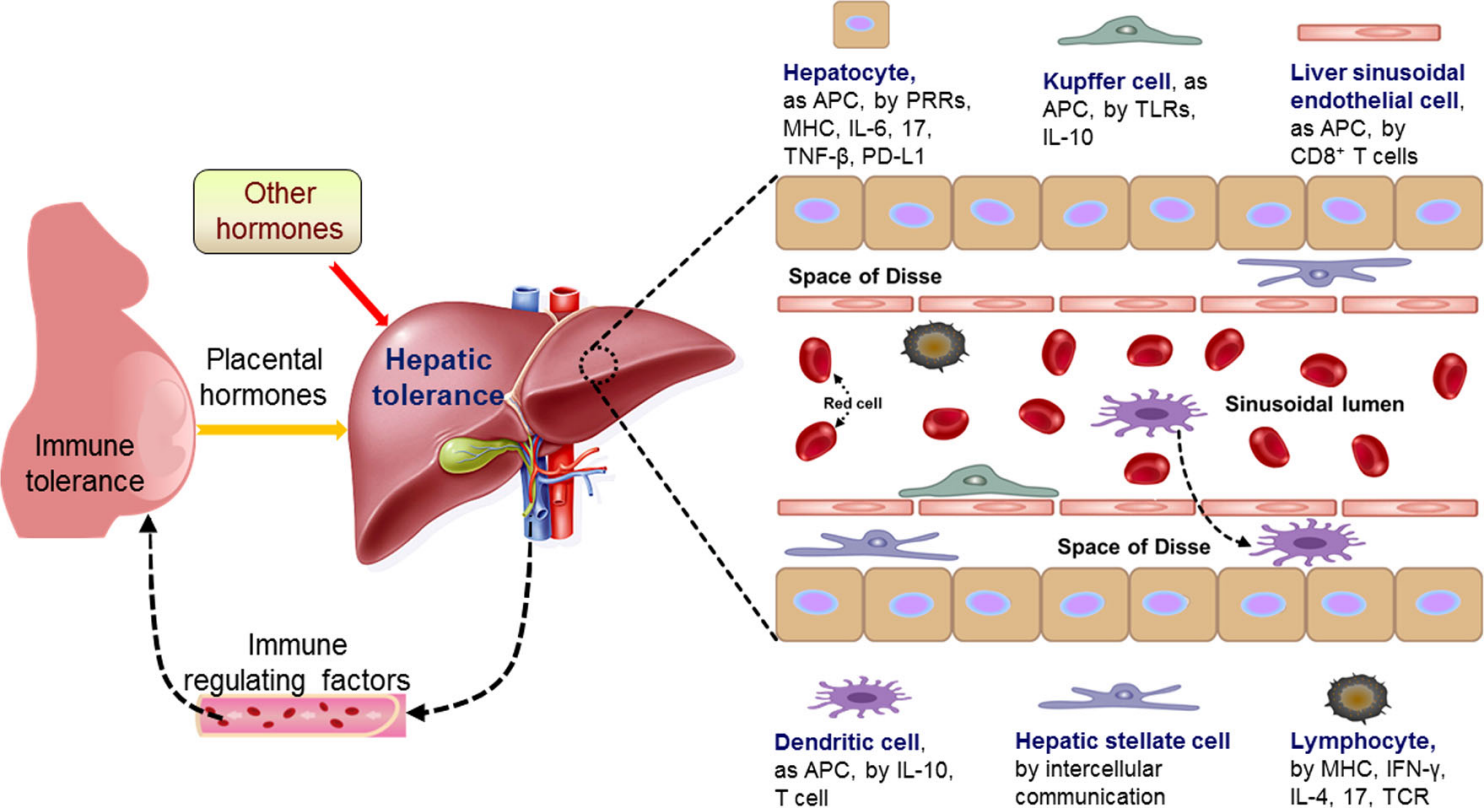
Pregnancy modulates maternal hepatic immune tolerance by placental hormones and other hormones. During pregnancy, the immune functions of maternal hepatic cells are modulated by placental hormones and other hormones, which lead to hepatic tolerance based on the recent advances. The changed immune regulating factors from the liver modulate maternal immune tolerance through blood circulation. Hepatocytes as antigen-presenting cells (APCs), are involved in regulating hepatic immune tolerance by modulation of pattern recognition receptor (PRRs), major histocompatibility complex (MHC), interleukin-6 (IL-6), IL-17, tumor necrosis factor (TNF)-β and programmed death ligand 1 (PD-L1), and kupffer cells, as APCs, are by regulation of toll-like receptors (TLRs) and IL-10. Furthermore, liver sinusoidal endothelial cells, as APCs, participate in hepatic immune tolerance by activation of CD8^+^ T cells and dendritic cells, as APCs, are implicated in hepatic immune tolerance by IL-10 and T cell. Moreover, hepatic stellate cells are by intercellular communication, and lymphocytes are by MHC, interferon-gamma (IFN-γ), IL-4, IL-17 and T cell receptor (TCR). Liver DCs can transverse the LSECs, enter the space of Disse.

In the liver, sinusoidal blood is actively scanned by a dense network of intravascular macrophages (such as KCs), a monolayer of LSECs, dendritic cells (DCs), hepatic stellate cells (HSCs), and liver-resident lymphocytes, including NKT cells, NK cells, and γδ-T cells, and these cells can activate and initiate the immune response through the production of cytokines and the recruitment of additional immune cells (16). The hepatic immunological structure mainly includes hepatocytes, HSCs, LSECs, KCs, DCs and lymphocytes, which are involved in the modulation of maternal immune responses (**Figure 2**).

### 4.1 Hepatocytes

The primary roles of hepatocytes are metabolism, protein production (including immune proteins), and toxin neutralization, and these cells express a wide variety of immune receptors (including pattern recognition receptor and major histocompatibility complex (MHC)) that are involved in hepatocyte-mediated immunity and the adaptive immune response (17, 18). Studies have shown that hepatocytes act as antigen-presenting cells (APCs) to activate naive CD4^+^ or CD8^+^ T cells (19, 20). In addition, proinflammatory hepatocytes are implicated in the recruitment of macrophages, and the recruited macrophages release cytokines (including IL-6, TNF-β, and IL-17) to induce the expression of inhibitory ligands, such as programmed death ligand 1 (PD-L1), in hepatocytes (21). Furthermore, hepatocytes produce a wide variety of innate immune proteins, including liver-enriched transcription factors and proinflammatory mediators, which modulate the expression of these innate immune proteins (22). Therefore, hepatocytes are implicated in the maternal adaptive immune response.

### 4.2 Kupffer cells

KCs are the largest population of liver-resident macrophages, and fill the sinusoidal lumens to scan for any foreign material in the blood. KCs express Toll-like receptors (TLRs), complement receptors, antibody receptors, and molecules that are involved in detecting, binding and internalizing pathogens, and activating KCs (1).

KCs can act as incompetent APCs, and suppress T-cell activation induced by other potent APCs, contributing to liver-mediated systemic immune tolerance (23). In addition, hepatic regulatory T cells (Tregs) and KCs prevent the establishment of the cytotoxic T lymphocyte response by inducing the secretion of the immunosuppressive cytokine IL-10 and create a local suppressive microenvironment in the liver (24). Moreover, KCs clear activated self cells, limit the potential of these cells to secrete inflammatory mediators, and participate in regulating inflammation (1). Therefore, KCs are involved in hepatic immunoregulation by producing immune-related cytokines and chemokines and cooperating with other innate immune cells.

### 4.3 Liver sinusoidal endothelial cells

LSECs form the wall of the hepatic sinusoids and play key roles in the maintenance of hepatic homeostasis and control of the hepatic immune response (25). LSECs have anti-inflammatory and anti-fibrogenic properties by inhibiting KC and HSC activation, and regulating intrahepatic vascular resistance and portal pressure (26). In addition, LSECs not only present endogenous antigens to CD8^+^ T cells but also efficiently function as true APCs in the cross-presentation of exogenous antigens. Moreover, LSECs can cross-present soluble exogenous antigens in the circulation to CD8^+^ T cells, leading to CD8^+^ T-cell tolerance rather than immunity (27). Therefore, LSECs act as APCs and modulate KC and HSC activation to participate in hepatic immunoregulation.

### 4.4 Liver dendritic cells

Liver DCs are derived from the bone marrow and are typically located around the central veins and portal tracts (16). DCs are the major APCs within all tissues of the body, and liver DCs can serve as a priming site for liver-infiltrating T cells in response to inflammation (28, 29). In addition, liver DCs internalize antigens derived from the blood, but DCs are poor APCs and promote tolerance rather than the activation of T cells under basal conditions (1). Furthermore, liver DCs mature during intrahepatic translocation from the portal circulation to the central vein (30), and liver DCs can transverse LSECs, enter the space of Disse and exit the liver *via* lymphatic drainage (31).

Liver DCs can be broadly divided into myeloid DCs (mDCs), plasmacytoid DCs (pDCs) and other DCs, and mDCs are further defined as CD11b^+^ and CD11c^+^ cells in mice (32). In contrast to mDCs and pDCs in the liver, CD11c^+^CD8^+^ DCs support robust T-cell activation, leading to the generation of T helper 1 (Th1)-type responses (29, 33). Furthermore, the suppression of immunosuppressive signals, such as the blockade of IL-10, can improve the development of DCs that enhance antigen presentation, costimulatory molecule expression, and T-cell activation in the liver (34). Therefore, liver DCs regulate hepatic immune tolerance by modulating T-cell activation depending on signals from the local environment.

### 4.5 Hepatic stellate cells

HSCs reside in the space of Disse, have an astral phenotype and play central roles in liver development, hepatocyte homeostasis, and the coordinated response of the liver to injury (35). There is intercellular communication *via* soluble mediators and cytokines/chemokines between HSCs and neighboring cell types, including hepatocytes, biliary epithelial cells, hepatic progenitor cells, KCs, bone marrow-derived macrophages, LSECs, infiltrating immune cells, and nerve cells (29). HSCs can be activated by many signaling molecules and convergent pathways that include fibrogenic, proliferative, and inflammatory cytokines, hedgehog signaling, metabolic reprogramming, cholesterol signaling, and oxidative stress. Furthermore, cytokines and growth factors secreted by hepatic immune cells are the most potent activating signals for HSCs, and activated HSCs express innate immune receptors related to TLRs and the complement pathway that are direct activators of HSCs (35). Therefore, hepatic immune cells secrete cytokines and growth factors that activate HSCs in a paracrine manner, which regulate hepatocyte homeostasis and the adaptive response.

### 4.6 Liver-resident lymphocytes

The liver contains both resident and transiting lymphocytes. There are multiple types of liver-resident lymphocytes, including memory CD8^+^ T cells, unconventional T cells (invariant NKT cells, mucosal-associated invariant T-cells, and γδT cells), NK cells and other innate lymphoid cells (36). CD8^+^ T cells are enriched in the liver, and the largest population of γδ T cells is in the liver. In addition, nearly half of lymphocytes in the liver express the T-cell receptor (TCR) (37). Furthermore, γδ T cells are distinct from conventional T-cells and can bind to several different foreign and self ligands in both MHC-dependent and MHC-independent manners, contributing to the immune response and immunopathology (38).

Liver NK cells express a restricted TCR repertoire and respond to lipid antigens to directly kill target cells *via* perforin and granzyme. In addition, NK cells can initiate both inflammatory and anti-inflammatory responses and produce a wide array of cytokines, including interferon-gamma (IFN-γ), IL-4 and IL-17 (39). There is an expanded population of liver-resident CD4^+^ T cells in patients with primary sclerosing cholangitis (PSC) compared to healthy individuals, and naive CD4^+^ T cells in PSC patients trend to develop into Th17 cells (40). Therefore, liver-resident lymphocytes produce cytokines that modulate the hepatic immune response and immunopathology, as well as systemic immune status.

## 5 Immunological adaptations in the maternal liver

In the liver, self and foreign antigens are presented by DCs, LSECs, KCs, HSCs and hepatocytes, which induce the expression of anti-inflammatory mediators and inhibitory cell surface ligands associated with T-cell activation, and contribute to systemic tolerance and the local immunosuppressive milieu (41). In addition, hepatic microenvironmental factors induce the expression of tolerogenic human leukocyte antigen G proteins in hepatic immune cells, hepatocytes and liver epithelial cells, which are associated with the tolerogenic properties of the liver (42). Furthermore, as a type-1 membrane glycoprotein, CD200 contains two immunoglobulin superfamily domains, is expressed in hepatic endothelial structures, and plays a pivotal role in the systemic immune tolerogenic effect of the liver (43). On the other hand, trophoblasts express CD200, which counter-regulates the Th1-type proinflammatory cytokine-inducible fibrinogen-related prothrombinase fgl2. However, fgl2 can terminate pregnancy *via* thrombin activation, and CD200-dependent downregulation of the Th1-type cytokine response is related to maternal hepatic tolerance during pregnancy (44). Moreover, acetaminophen-induced liver inflammation interferes with maternal immune and endocrine adaptation, which increases plasma alanine aminotransferase, decreases plasma progesterone, and morphologically alters the placenta (45).

There are increases in the expression of interferon-stimulated gene 15-kDa protein (ISG15) and myxovirus resistance protein 1 (Mx1) in the bovine liver during the late peri-implantation period (46). Our previous studies showed ISG15 and ISG15-conjugated proteins are upregulated in the ovine liver during early pregnancy. In addition, 2’,5’-oligoadenylate synthetase 1, Mx1, interferon-gamma-inducible protein 10 and STAT1 are increased in the maternal liver during early pregnancy (47).

**Figure 3:**
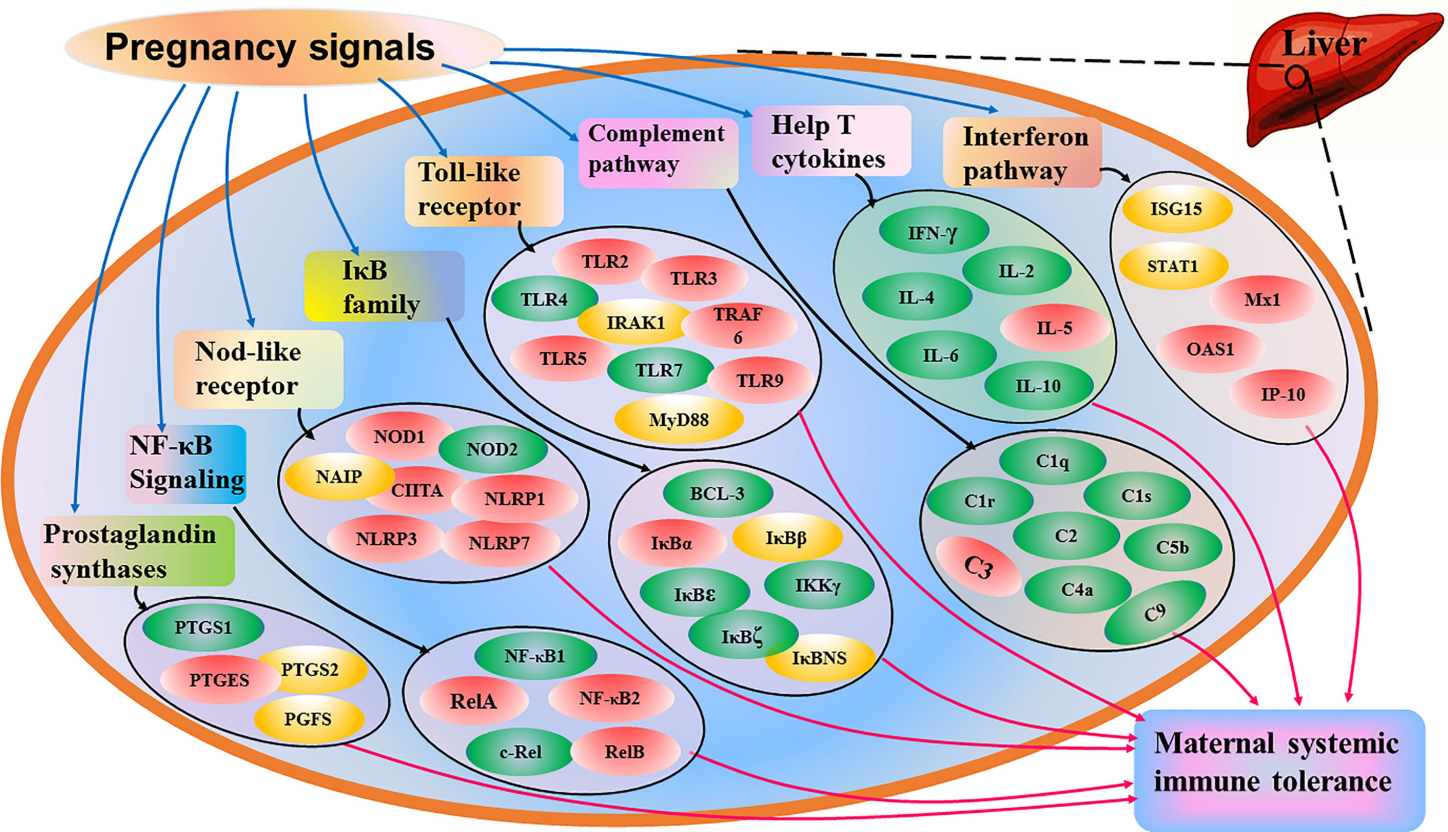
Sketch of immunological adaptations in maternal liver during pregnancy based on the recent advances. Pregnancy signals induce changed expression of interferon pathway, help T cytokines, complement pathway, toll-like receptor, IkappaB (IκB) family, nuclear factor kappaB (NF-κB) signaling, nod-like receptor and prostaglandins E synthase, which contributes to maternal systemic immune tolerance during pregnancy. Interferon pathway includes interferon-stimulated gene 15 kDa protein (ISG15), signal transducer and activator of transcription 1 (STAT1), myxovirusresistance protein 1 (Mx1), 2’,5’-oligoadenylate synthetase 1 (OAS1) and interferon-gamma-inducible protein 10 (IP-10), and help T cytokines include interleukin (IL)-2, interferon-gamma (IFN-γ), tumor necrosis factor (TNF)-β, IL-4, IL-5, IL-6 and IL-10. Complement pathway includes complement components C1q, C1r, C1s, C2, C3, C4, C5 and C9, and toll-like receptors (TLRs) include TLR2, TLR3, TLR4, TLR5, TLR7, TLR9, myeloid differentiation primary-response protein 88 (MyD88), tumor necrosis factor receptor associated factor 6 (TRAF6) and interleukin-1-receptor-associated kinase 1 (IRAK1). IkappaB (IκB) family includes B cell leukemia-3 (BCL-3), IκBα, IκBβ, IκBϵ, IKKγ, IκBζ and IκBNS, and nuclear factor kappaB (NF-κB) signaling includes NF-κB1, NF-κB2, RelA, RelB and c-Rel. Nucleotide-binding oligomerization domain (NOD) receptors (NLRs) NOD1, NOD2, major histocompatibility complex class II transactivator (CIITA), neuronal apoptosis inhibitor protein (NAIP), NLR family, pyrin domain-containing 1 (NLRP1), NLRP3 and NLRP7, and prostaglandin synthases include prostaglandin-endoperoxide synthase 1 (PTGS1), PTGS2, PGE synthase (PTGES) and PGF synthase (PGFS). Note: red, stimulators; green, negative regulators; yellow, changed.

Our previous studies showed that early pregnancy regulates the expression of Th cytokines in the ovine maternal liver, including IL-2, IFN-γ, TNF-β, IL-4, IL-5, IL-6, and IL-10 (48), and the TLR pathway is modulated in the ovine maternal liver during early pregnancy (49). The expression of nuclear factor kappaB (NF-κB) signaling in the ovine maternal liver, including NF-κB1 (p50), NF-κB2 (p52), RelA (p65), RelB and c-Rel, is modulated during early pregnancy in sheep (50). In addition, early pregnancy modulates the expression of the IkappaB (IκB) family, including B-cell leukemia-3 (BCL-3), IκBα, IκBβ, IκBϵ, IKKγ, IκBNS and IκBζ in the livers of ewes (51). Furthermore, the expression of complement components in the maternal liver, including C1q, C1r, C1s, C2, C4a, C5b and C9, is changed in ewes (52). Moreover, the expression of CD4 is improved, and nod-like receptor expression is regulated in the maternal liver during early pregnancy in sheep (53, 54). Therefore, pregnancy modulates the expression of many cytokines and signaling pathways, which contribute to systemic tolerance and the local immunosuppressive milieu in the liver (**Figure 3**). However, more studies are needed to confirm whether other cytokines and signaling pathways are associated with maternal hepatic adaptations during pregnancy.

## 6 Maternal pregnancy-associated liver diseases

**Figure 4:**
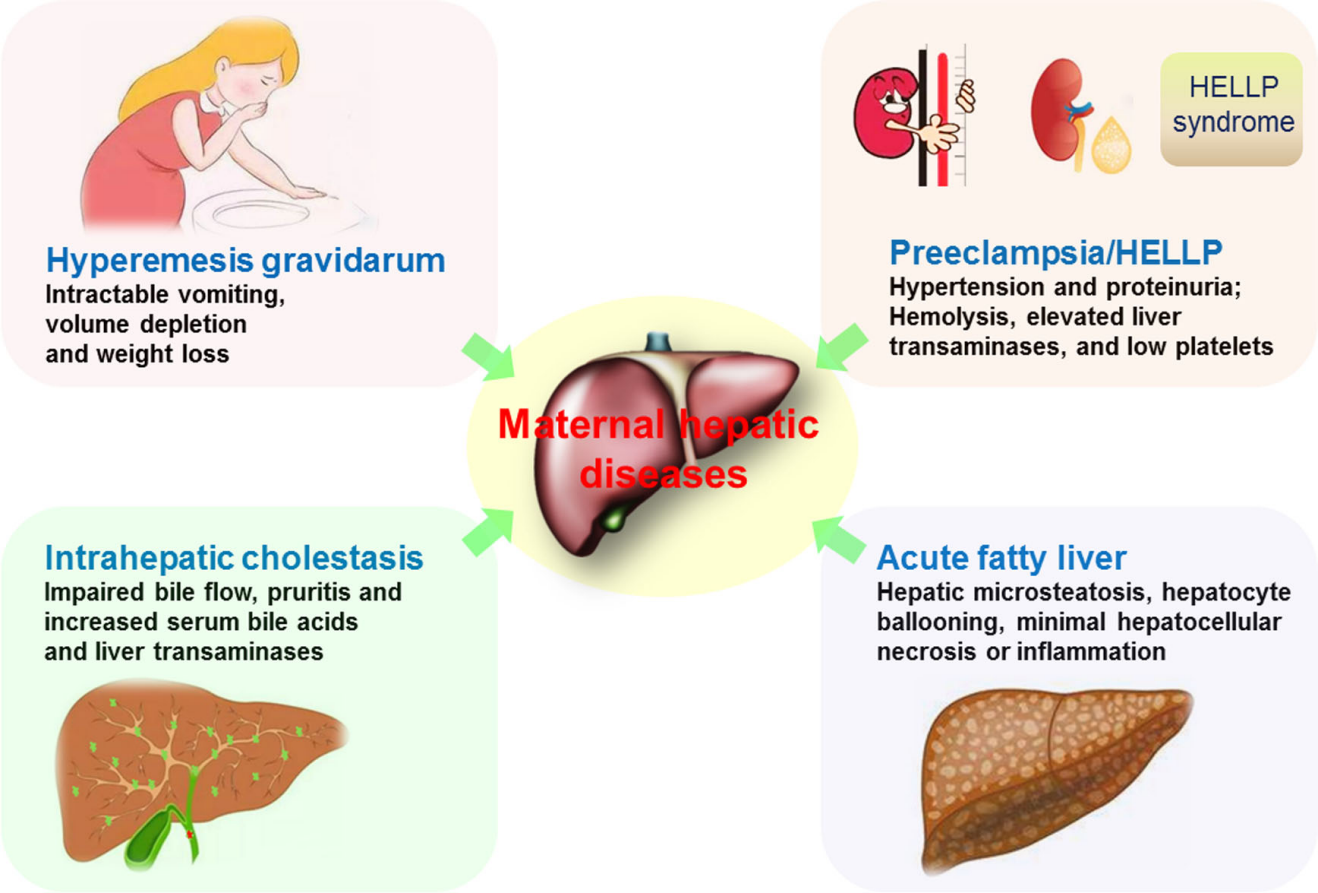
Maternal pregnancy-associated liver diseases. Hyperemesis gravidarum is described as intractable vomiting that leads to volume depletion and weight loss. Intrahepatic cholestasis has the impaired bile flow that results in pruritis and increases in serum bile acids and liver transaminases. Preeclampsia presents hypertension and proteinuria, and HELLP is associated with preeclampsia, characterized by hemolysis, elevated liver enzymes, and low platelet count syndrome. Acute fatty liver of pregnancy is characterized by hepatic microsteatosis, hepatocyte ballooning, minimal hepatocellular necrosis or inflammation.

There are four main pregnancy-associated liver diseases that include hyperemesis gravidarum (HG); intrahepatic cholestasis of pregnancy (ICP); liver disorders associated with preeclampsia, specifically hemolysis, elevated liver enzymes, and low platelet count syndrome (HELLP); and acute fatty liver of pregnancy (AFLP) (**Figure 4**). These liver diseases are closely associated with significant maternal and fetal/neonatal morbidity and mortality (55). Inflammation induced by environmental factors and general stress leads to defects in immune tolerance of the maternal liver. The local and systemic immune tolerance is related to liver resident nonconventional APCs, including LSECs, HSCs and hepatocytes (44).

### 6.1 Hyperemesis gravidarum

HG is described as intractable vomiting during pregnancy, results in volume depletion and weight loss, and starts at 4 or 5 weeks during the first trimester of pregnancy (56). HG has a multifactorial etiology, including molar pregnancy, multifetal pregnancy, hypoadrenalism, hyperthyroidism, cerebral malignancy, and gastrointestinal obstruction (55).

The causes of HG are related to hormones in maintaining pregnancy, and the level of human chorionic gonadotropin (hCG) correlates closely with HG during pregnancy (57). Ketonuria levels and inflammatory markers platelet-to-lymphocyte ratio, monocyte-to-lymphocyte ratio, and neutrophil-to-lymphocyte ratio are higher in pregnant patients with HG (58). Th1 and Th2 cytokines, estrogen, progesterone receptor, growth/differentiation factor 15 and its receptor, glial-derived neurotrophic factor family receptor alpha-like are closely related to HG (44, 56). Th cytokines, including IFN-γ, IL-2, IL-4, IL-6 and IL-10, decrease, but IL-5 increases in the maternal liver during early pregnancy in an animal model (48). Progesterone receptor expression level upregulates in the maternal liver during early pregnancy, which is associated with the modulation of maternal hepatic immune function in an animal model (59).

The treatment for HG includes pharmaceutical treatment and replenishing lost nutrients. Pharmaceutical treatment includes antiemetics plus correcting electrolytes, as well as intravenous fluids and considering admission if needed (44). The prevention includes nutritional support, such as vitamin replacement, and enteral tube feeding (55). Further study should focus on establishing underlying etiology for designing individualized management strategies, and long-term safety of new therapies for the mother.

### 6.2 Intrahepatic cholestasis of pregnancy

ICP is defined as impaired bile flow, the accumulation of bile constituents in the liver and blood, which causes pruritus and increases in serum bile acids and liver transaminases, and is also related to fetal distress, spontaneous and iatrogenic preterm birth, and stillbirth (60). ICP starts at the last trimester of pregnancy in the majority of patients, and also possibly onsets earlier (44).

The causes of ICP are related to mechanical obstruction and deficiency in cellular transporters. The familial cholestasis type 1, bile salt export pump and multidrug resistance p-glycoprotein 3, ABC transporter superfamily, vascular endothelial growth factor, estrogen, MHC class I molecule, vascular cell adhesion molecule, Th cytokines are closely associated with ICP (44, 61). In addition, peroxisomal acyl-CoA oxidase 1, L-palmitoylcarnitine, and glycocholic acid expression levels in placental and serum are significantly higher in third-trimester ICP patients (62).

The mainly treatment for ICP is with ursodeoxycholic acid (UDCA) that is a bile acid. UDCA treatment reduces spontaneous preterm birth in ICP, in particular in singleton pregnancies with maternal serum bile acid concentrations 40 μmol/L or more, but has no significant effect on the prevalence of stillbirth in women with ICP (63). Rifampicin combines with UDCA treatment reduces pruritus and serum bile acid concentration, and Rifampicin can improve cholestatic pruritus in nonpregnant individuals (55). Studies are needed for the future on improving pregnancy outcome and maternal pruritus using new therapies, and identifying women at risk of future hepatobiliary disease *via* genetic tests.

### 6.3 Preeclampsia/HELLP

The preeclampsia is definited as the presence of hypertension and proteinuria occurring after 20 weeks of gestation in a previously normotensive patient. Systemic manifestations of preeclampsia include low platelets or elevated liver enzymes, and preeclampsia also results in other maternal organ dysfunction and fetal growth restriction (64). HELLP syndrome is a pregnancy-associated liver disease, and termed as a severe complication of preeclampsia.

The causes of preeclampsia/HELLP are mainly related to abnormal placentation, trophoblast invasion and maternal-fetal interface (65), which result in increased release of inflammatory mediators into the maternal circulation, imbalance between pro- and anti-angiogenic factors, liver and vascular dysfunction, damaged endothelium, vasospasm, platelet aggregation, endothelial damage and hemolytic anemia (55). There are many cytokines that are related to preeclampsia/HELLP, including TNF-α, transforming growth factor-β, a disintegrin and metalloproteases with thrombospondin type 1 motif 12, IL-1 family, IL-17, IL-18-binding protein, IL-33, intercellular adhesion molecule-1, monocyte chemoattractant peptide 1, pentraxin 3, soluble vascular adhesion molecule 1, C reactive protein, soluble fms-like tyrosine kinase-1, vascular endothelial growth factor A, pregnancy-associated plasma protein A2, aspartate aminotransferase, alanine aminotransferase, Th cytokines, soluble Fas ligand, soluble CD40 ligand (44).

The mainly treatment for preeclampsia is with low dose prophylactic aspirin, MgSO_4_, oral calcium, vitamin D, melatonin, detoxification of reactive oxygen species, and treatment for HELLP with corticosteroid (44). It is reported that prophylactic low-dose aspirin can reduce the risk of preterm preeclampsia, but no drugs can influence disease progression except for delivery (66). Future researches needed include identification of new and refinement of biomarkers for predicting preeclampsia, and evaluation of hemostasis markers of acute liver injury for aiding the management of bleeding/thrombosis complications in HELLP.

### 6.4 Acute fatty liver of pregnancy

AFLP is definited as jaundice with rapidly progressive liver failure in the third trimester of pregnancy, and characterized by hepatic microsteatosis, hepatocyte ballooning, minimal hepatocellular necrosis or inflammation (55).

The causes of AFLP are associated with long-chain acyl-coenzyme A dehydrogenase deficiency, mitochondrial dysfunction and abnormal β-oxidation of fatty acids long-chain fatty acid (44). In addition, the maternal characteristics, including first pregnancy, multifetal pregnancy, male fetus, and low body mass index, are related to AFLP (55).

The mainly treatment for AFLP is with intensive care treatment, including plasma exchange, plasma perfusion, or a combination of both. Immediate delivery is also a choice in most cases, and severe cases may require liver transplantation (44). There are infectious complications in AFLP, so low dose antibiotics treatment is recommended (55). Further studies are needed in identification of biomarkers of at-risk pregnancies to minimize maternal and fetal complications, and delineation of risk factors, such as environmental risks, maternal conditions and family history.

## 7 Factors that regulate hepatic immunology

**Figure 5:**
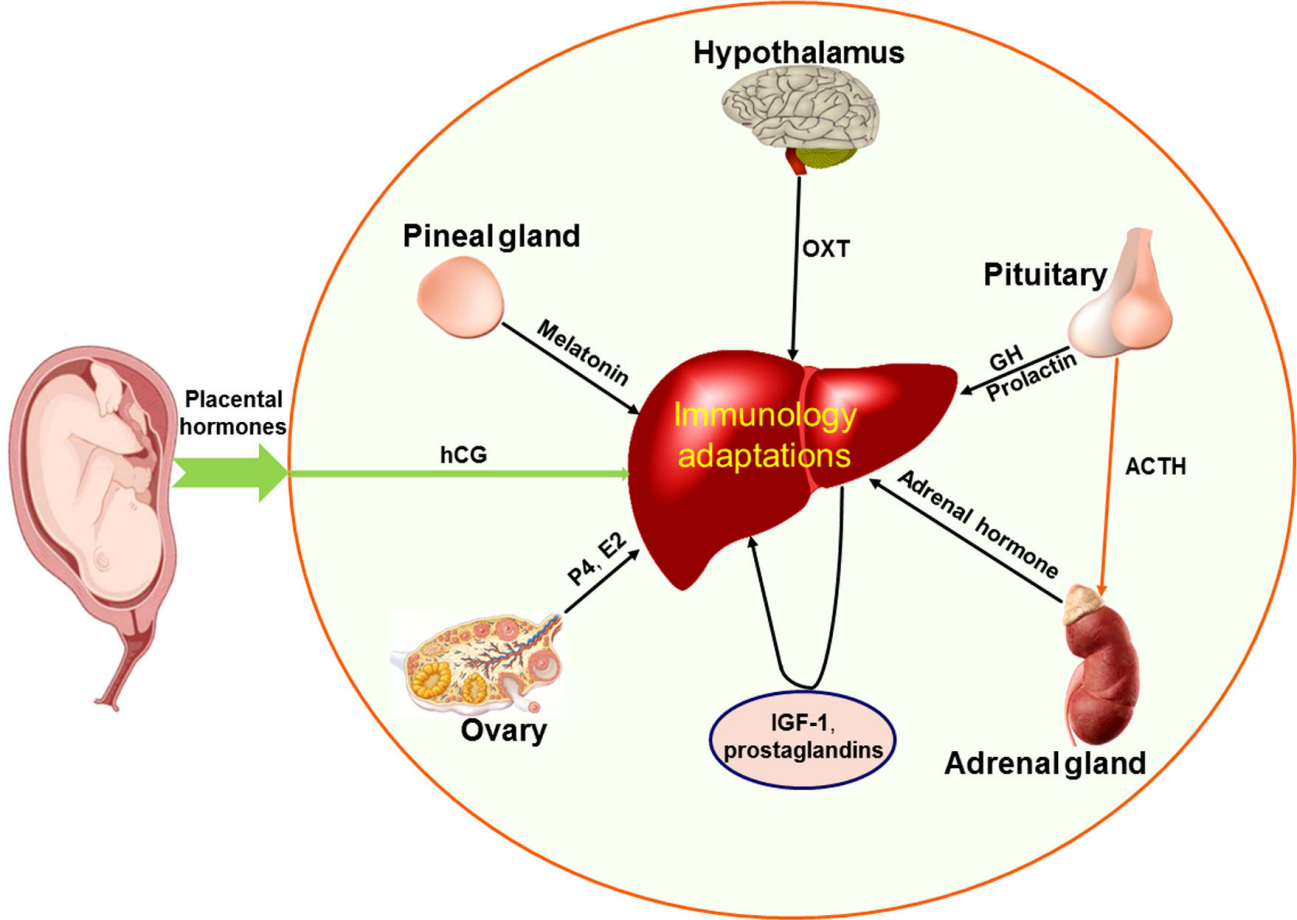
Factors that regulate maternal hepatic immunological adaptations during pregnancy based on the recent advances. During pregnancy, placental hormones (including human chorionic gonadotropin, hCG) have effects on the liver, ovaries, hypothalamus, pituitary gland, pineal gland and adrenal glands, which participate in regulating maternal hepatic immunological adaptations during pregnancy. Other hormones and hCG directly modulate the hepatic immunology. The other hormones include estrogen (E2) and progesterone (P4) mainly produced by ovaries, insulin like growth factor 1 (IGF-1) and prostaglandins produced by the liver in a paracrine manner, oxytocin (OXT) produced by hypothalamus, growth hormone (GH), adrenocorticotropic hormone (ACTH) and prolactin produced by pituitary gland, melatonin secreted by the pineal gland, and adrenal hormone mainly produced by adrenal glands.

Maternal endocrine hormone profiles are modulated during pregnancy, and these hormones include steroid hormones, growth hormone (GH)/insulin-like growth factor 1 (IGF-1), prolactin, adrenocorticotropic hormone (ACTH), human chorionic gonadotropin (hCG), metanephrines, and aldosterone (67). In summary, the factors that regulate hepatic immunology include hCG, estrogen, progesterone, GH/IGF-1, prolactin, ACTH, aldosterone, adrenaline, melatonin, oxytocin and prostaglandins (PGs), which are implicated in the regulation of hepatic immunological adaptations during pregnancy (**Figure 5**).

### 7.1 Human chorionic gonadotropin

The placenta secretes hCG, which has a series of effects on the survival of the embryo during pregnancy (68). It has been reported that chorionic gonadotropin can induce maternal hepatocyte proliferation during early pregnancy in mice (69). Silent mating type information regulation 2 homolog 1 and forkhead box o3 axis in hepatocytes is regulated by hCG, which participates in immune suppression, and has therapeutic effects on inflammatory liver diseases (70). However, hCG is also closely related to HG during pregnancy (57). Therefore, the therapeutic effects of hCG on pregnancy-associated liver diseases need further study.

### 7.2 Estrogen

Serum estrogen levels increase during normal pregnancy (71), and estrogen stimulates hepatocyte proliferation in late pregnancy in mice (67). In addition, significant increases in estrogen increase CD4^+^ CD25^+^ Treg cells in peripheral blood to influence the immune function of pregnant women (72). Estrogen signaling is related to hepatic oxidative damage and the expression level of peroxisome proliferator-activated receptor-γ (PPARG) coactivator 1α (PGC1A) in the mouse liver, and these factors are involved in steatohepatitis (73). However, estrogen has protective effects on liver diseases, including nonalcoholic fatty liver disease (NAFLD) and nonalcoholic steatohepatitis (NASH) (74). Therefore, further investigations may focus on the effects of estrogen on liver health during normal pregnancy.

### 7.3 Progesterone

Serum progesterone levels are upregulated during normal pregnancy, and the immune system is modulated by circulating progesterone, which plays a key role in halothane-induced liver injury *via* immune-related responses (75). Progesterone is related with decreases in the inflammatory reactions, the activation of immune cells and the production of cytokines, which are essential for the successful pregnancy (76). Our recent study showed that progesterone receptor and progesterone-induced blocking factor were upregulated in the ovine liver during early pregnancy, which is involved in the regulation of maternal hepatic immune and other functions in an endocrine manner (59). Progesterone can increase hepatitis E virus (HEV) replication and results in severe HEV through activation of the nonclassical progesterone receptor membrane component 1/2 signaling pathway, rather than immunomodulation of HEV-induced interferon response (77). Vaginal progesterone levels are positively related to ICP, and progesterone metabolites are increased during pregnancy, which enhances the risk of ICP (78). Progesterone use is related to more severe lobular inflammation and influences NAFLD pathobiology (79). High levels of progesterone are favorable for pregnancy maintenance but are also the main cause of maternal pregnancy-associated liver diseases. Therefore, the effects of progesterone on liver health should be considered in future studies.

### 7.4 Growth hormone/insulin like-growth factor 1

GH produced by the pituitary gland regulates the production of IGF-1 by the liver, and its effects are directly mediated by the GH receptor (GHR). There is an inverse association between hepatic expression of the IGF system (IGF-1 and GHR) and certain cytokines (IL-1β, IL-18 and TNF-α) and acute-phase proteins (serum amyloid A and haptoglobin) in the liver, which are associated with hepatic immunomodulation in pigs (80). In addition, GH activates c-JUN to increase the induction of the regulator of calcineurin 1-4, which affects the activity of calcineurin in the rat liver and participates in T-cell activation and gluconeogenesis (81). Furthermore, IGF-1 inhibits the expression of TNF-α, IL-1β, and cytokine-induced neutrophil chemoattractant 1 and accelerates the degradation of inducible nitric oxide synthase caused by D-galactosamine and lipopolysaccharide to prevent liver injury through an NF-κB-independent pathway in rats (82). GH replacement therapy improves NAFLD/NASH, and IGF-I treatment can improve NASH and cirrhosis in animal models (83).

Hepatic GH-binding protein and GHR mRNA levels are upregulated during pregnancy (84), but the expression levels of hepatic GHR 1A and IGF-1 mRNA are downregulated at parturition in cows (85). In addition, the expression level of IGF binding protein 4 (IGFBP-4) mRNA is increased in the maternal liver, but the expression level of IGFBP-3 mRNA is decreased during pregnancy in rats (86). Therefore, GH and IGF-1 are involved in the regulation of maternal liver function during pregnancy and have therapeutic effects on liver diseases.

### 7.5 Prolactin

Prolactin is primarily synthesized in the anterior pituitary gland, as well as the central nervous system, the immune system, and the uterus (87). In addition, prolactin improves normal liver growth, survival, and regeneration in prolactin receptor (PRLR)-dependent and/or independent manners, which are associated with hepatic IL-6, a suppressor of cytokine signaling-3 in rodents (88). Furthermore, prolactin suppresses mitogen-activated protein kinase-dependent activation of c-Myc *via* PRLR to prevent tumor-promoting liver inflammation in mice (89).

Our recent study showed that early pregnancy inhibited the expression of prolactin and PRLR in the liver in ewes, and PRLR protein was located in hepatocytes, endothelial cells of the proper hepatic arteries and hepatic portal veins (90). PRLR is suppressed in mammary glandular tissue during periods of high progesterone levels and increases when the serum progesterone level drops in mice (78). Prolactin ameliorates hepatic steatosis *via* PRLR and fatty acid translocase, which can be used to improve NAFLD (91). Therefore, prolactin has therapeutic effects on liver diseases, but the use of prolactin in liver disease therapy during pregnancy should be done with caution.

### 7.6 ACTH, aldosterone and adrenaline

Adrenal glands mainly produce corticosteroid-, aldosterone-, cortisol- and adrenaline-type hormones. Aldosterone promotes caveolin 1-related selective autophagy and F-actin remodeling by activating the AMP-activated protein kinase (AMPK)-ULK1 pathway and inhibiting the NO-dependent pathway, which induces LSEC defenestration *via* aldosterone-induced oxidation (92). In addition, aldosterone induces the activation of primary mouse HSCs by promoting the expression and assembly of the NOD-like receptor (NLR) family, pyrin domain containing 3 inflammasome and liver fibrosis (93). Mineralocorticoid receptor (MR) is activated by aldosterone or glucocorticoids, as well as pathological milieu factors, and associated with the development of NAFLD and NASH (94). Treatment with eplerenone, an MR antagonist, inhibits the development of fibrosis and portal hypertension related complications in an animal model (95). However, effects of ACTH, aldosterone and adrenaline on the maternal liver during pregnancy should be investigated.

### 7.7 Melatonin

Melatonin is secreted by the pineal gland, and acts directly on the liver to elevate plasma glucose levels *via* melatonin receptor 1B in mouse hepatocytes in a dose-dependent manner (96). Prenatal glucocorticoid overexposure results in maternal hepatic steatosis, the downregulation of leptin and the upregulation of caspase 3 and TNF-α proteins levels, DNA methyltransferase activity and DNA methylation, and melatonin can rescue these adverse effects in the rat liver (97).

Serum melatonin levels are significantly lower in women with ICP than in healthy pregnant women (98). Our study showed that melatonin receptor 1A was upregulated in the maternal liver, but melatonin receptor 1B is downregulated during early pregnancy in sheep (54). Sato et al. showed melatonin administration not only had antioxidant effects, but also attenuated liver steatosis in NAFLD, increased functions and enzyme activity in mitochondria to inhibit lipid accumulation in the liver (99). Therefore, the use of melatonin in maternal pregnancy-associated liver disease therapy should be explored.

### 7.8 Oxytocin

Oxytocin is mainly produced by the hypothalamus, and oxytocin treatment modulates hepatic immune and inflammatory processes to protect against sepsis-induced oxidative damage (100). Oxytocin is synthesized by magnocellular neurons, and oxytocin secretion is upregulated by intracerebroventricular kisspeptin in late pregnancy (101). In addition, oxytocin treatment induces thermogenic gene expression, which ameliorates fatty liver in an animal model (102). Therefore, more studies may focus on the use of oxytocin to treat maternal pregnancy-associated liver disease.

### 7.9 Prostaglandins

PGs can suppress generation of the reactive oxygen species, modulate production of the inflammatory cytokines and cell adhesion molecules, which have protective effects on ischemia reperfusion-injured livers (103). Intraportal PGE1 infusion can protect extrahepatic cholestatic liver from ischemia reperfusion injury by reducing oxidative stress damage, intrahepatic neutrophil infiltration and hepatocyte apoptosis in rat model of extrahepatic cholestasis (104).

Mesenchymal stem cell (MSC) treatment enhances YAP and β-catenin expression with increasing PGE2 production in the ischemic livers, indicating that PGE2 is related to MSC-mediated immunotherapy of liver sterile inflammatory injury (105). PGE2 levels are higher throughout late pregnancy in mice, and high levels of PGE2 during late pregnancy protect fetuses from inflammatory damage related to IL-1β (106). In addition, prostaglandin-endoperoxide synthase 2, PGE synthase and PGF synthase levels are upregulated in the ovine maternal liver during early pregnancy, which are related to the hepatic immune adjustment during early pregnancy in sheep (107). Therefore, PGs could be used to treat liver disease, but more studies are needed to confirm this use during pregnancy.

## 8 Conclusion

Pregnancy changes the expression of many cytokines and signaling pathways (**Figure 3**), which are related to the maintenance of maternal liver homeostasis and immune tolerance, as well as pregnancy-associated diseases. Maternal immunological adaptations in the liver are necessary for successful pregnancy and are directly associated with avoiding embryo loss and abortion. In addition, the liver disorders in pregnancy may be mainly associated with hCG, Th1 and Th2 cytokines, estrogen and progesterone. In this article, the factors that regulate hepatic immunology are discussed, which may be used to avoid detrimental immune responses against the allogeneic fetus and prevent maternal hepatic diseases related to pregnancy. However, many mysteries still exist regarding the modulation of hepatic immune functions during pregnancy. Therefore, future studies are needed in these areas, which will bring new hopes for the prevention of embryo loss and abortion during pregnancy, as well as pregnancy-associated immunological diseases.

## Author contributions

All authors contributed to the development of this review article and approved the submitted version. LY and YM searched the literature and drafted the manuscript. LY and YS conceived and designed the review. HF and LZ examined the literature and made the figures. LY made a critical revision of the review. All authors contributed to the article and approved the submitted version.
